# *Plasmodium chabaudi chabaudi *malaria parasites can develop stable resistance to atovaquone with a mutation in the cytochrome b gene

**DOI:** 10.1186/1475-2875-9-135

**Published:** 2010-05-21

**Authors:** Ana Afonso, Zoraima Neto, Helena Castro, Dinora Lopes, Ana C Alves, Ana M Tomás, Virgílio D Rosário

**Affiliations:** 1 Centro de Malária e Doenças Tropicais - Associated Laboratory (CMDT-LA)/IHMT/UEI Malaria, Rua da Junqueira 100, 1349-008 Lisbon, Portugal; 2 IBMC - Instituto de Biologia Molecular e Celular, Universidade do Porto, Rua do Campo Alegre 823, 4150-180 Porto, Portugal; 3 Unit of Medical Parasitology and Microbiology (UPMM)/IHMT Rua da Junqueira 100, 1349-008 Lisbon, Portugal; 4 Previously a member of Centro de Malária e Doenças Tropicais - Associated Laboratory (CMDT-LA)/IHMT/UEI Malaria, Rua da Junqueira 100, 1349-008 Lisbon, Portugal; 5 ICBAS - Instituto de Ciências Biomédicas Abel Salazar, Universidade do Porto, Portugal

## Abstract

**Background:**

*Plasmodium falciparum*, has developed resistance to many of the drugs in use. The recommended treatment policy is now to use drug combinations. The atovaquone-proguanil (AP) drug combination, is one of the treatment and prophylaxis options. Atovaquone (ATQ) exerts its action by inhibiting plasmodial mitochondria electron transport at the level of the cytochrome bc1 complex. *Plasmodium falciparum in vitro *resistance to ATQ has been associated with specific point mutations in the region spanning codons 271-284 of the *cytochrome b *gene. ATQ -resistant *Plasmodium yoelii *and *Plasmodium berghei *lines have been obtained and resistant lines have amino acid mutations in their CYT *b *protein sequences. *Plasmodium chabaudi *model for studying drug-responses and drug-resistance selection is a very useful rodent malaria model but no ATQ resistant parasites have been reported so far. The aim of this study was to determine the ATQ sensitivity of the *P. chabaudi *clones, to select a resistant parasite line and to perform genotypic characterization of the *cytb *gene of these clones.

**Methods:**

To select for ATQ resistance, *Plasmodium. chabaudi chabaudi *clones were exposed to gradually increasing concentrations of ATQ during several consecutive passages in mice. *Plasmodium chabaudi cytb *gene was amplified and sequenced.

**Results:**

ATQ resistance was selected from the clone AS-3CQ. In order to confirm whether an heritable genetic mutation underlies the response of AS-ATQ to ATQ, the stability of the drug resistance phenotype in this clone was evaluated by measuring drug responses after (i) multiple blood passages in the absence of the drug, (ii) freeze/thawing of parasites in liquid nitrogen and (iii) transmission through a mosquito host, *Anopheles stephensi*. ATQ resistance phenotype of the drug-selected parasite clone kept unaltered. Therefore, ATQ resistance in clone AS-ATQ is genetically encoded. The Minimum Curative Dose of AS-ATQ showed a six-fold increase in MCD to ATQ relative to AS-3CQ.

**Conclusions:**

A mutation was found on the *P. chabaudi cytb *gene from the AS-ATQ sample a substitution at the residue Tyr268 for an Asn, this mutation is homologous to the one found in *P. falciparum *isolates resistant to ATQ.

## Background

Malaria is one of the most common infectious diseases in the world and one of the greatest global public health problems, causing serious illness in 100-200 million people and estimated to kill two million people annually, with highest mortality rates among pregnant women and young children. A variety of drugs are available for therapy and prophylaxis. However, *Plasmodium falciparum*, the main malaria agent, has developed resistance to many of the drugs in common use (such as chloroquine and sulphadoxine) such that currently, drug combination therapy using atemisinin derivatives is the recommended treatment to prevent the appearance of multidrug-resistant parasites. Thus, the spread of resistant parasites is a serious health issue and new drugs are urgently needed.

The recommended treatment policy is now to use drug combinations [1]. The atovaquone (ATQ) -proguanil (AP) drug combination, distributed under the trade name of Malarone^®^, is one of the treatment and prophylaxis options. AP has a high cure rate, mild side effects [2], has potent blood schizonticidal activity and is also effective against the pre-erythrocytic and sexual stages and proved adequate in cases of multi-drug resistant *P. falciparum *malaria [3,4]. ATQ exerts its action by inhibiting plasmodial mitochondria electron transport at the level of the cytochrome bc1 complex leading to collapse of the mitochondrial membrane potential [4-8] while proguanil (an isopropylbiguanide) via its metabolite cycloguanil inhibits dihydrofolate reductase thus potenciating ATQ action [4,9,10].

*P. falciparum in vitro *resistance to ATQ has been associated with specific point mutations in the region spanning codons 271-284 of the *cytochrome b *gene (*Pfcytb*) [7,11,12]. A high frequency of recrudescence was observed in patients receiving ATQ, as a single drug therapy against *P. falciparum *[13,14]. A Y268S point mutation in the *Pfcytb *gene, distinct from the mutations observed in the lines selected *in vitro *for ATQ resistance, was detected in the recrudescing parasites [7]. The polymorphism in codon 268 is used as marker for a molecular surveillance of AP resistance [15-19]. AP treatment failures were increasingly reported a few years after its introduction, with recrudescing parasites presenting a markedly increased IC50 for ATQ [15,20,21]. In most cases, recrudescence is associated with a mutant 268 codon, either Y268S [3,12,20,22-26], Y268N [15] or Y268C mutation [21].

Along with the key issue of emergence and spreading of polymorphisms conferring ATQ resistance, analysis of *Pfcytb *field diversity presents an interest in population genetics [27-31]. Indeed, the *cytb *gene is encoded by the mitochondrial DNA and, as a consequence, is of uniparental inheritance and under different evolution constraints compared to nuclear genes [32,33]. In particular, interallelic recombination is not possible and polymorphisms, such as base substitutions or insertions, may accumulate over time. In mammals, the *cytb *locus displays an approximately 10-fold higher mutation rate than nuclear genes [34]. A rapid mutation rate (one mutation in 10^5 ^parasites) was reported for *Pfcytb *[35], but 100-1,000 lower rates were described subsequently [7].

A previous report showed that AP-resistant genotype of *P. falciparum *can be detected in non-exposed areas. Happi *et al *[36] found a Y268N mutation in *P. falciparum *from Nigeria, where AP had not been used; this finding suggests that the mutations in the *cytb *gene might occur naturally.

ATQ-resistant *Plasmodium yoelii *and *Plasmodium berghei *lines have been derived from infected mice treated with suboptimal doses of ATQ. All resistant lines have single or double amino acid mutations in their CYT *b *protein sequences [37,38], which were associated with increased resistance to the collapsing of mitochondrial membrane potentials and the inhibition of respiration afforded by ATQ, though these mutations are in different positions than the ones detected in resistant *P. falciparum*. Even though *Plasmodium chabaudi *is the most useful rodent malaria model with respect to drug responses, drug studies and drug resistance selection, no ATQ (ATQ) resistant parasites have been reported so far.

The aim of this study was to determine the ATQ sensitivity of the *P. chabaudi *clones, to select a resistant parasite line and to perform genotypic characterization of the *cytb *gene of these clones.

## Methods

### Parasites and hosts

*Plasmodium chabaudi chabaudi *clones were maintained in 4-6 week old laboratory CD1 female mice, as described by Walliker *et al *[39]. Mouse drinking water was supplemented with 0.05% paraminobenzoic acid (PABA).

The parasite clones used to select atovaquone (ATQ) resistance were pyrimethamine and chloroquine resistant mutants [40] derived from a cloned isolate, *P. c. chabaudi *clone AS-3CQ (See Table S1, Additional file 1). Briefly, the original AS isolate was cloned by limiting dilution (AS-SENS), then selected for resistance to pyrimethamine and recloned [41]. This clone, designated AS-PYR, was later selected for resistance to chloroquine and a line resistant to six daily doses of this drug at 3 mg kg-1 was obtained [42]. This line was cloned and denoted AS-3CQ. AS-3CQ was then subjected to further stepwise increases of chloroquine, producing a line resistant to six daily doses of chloroquine at 15 mg kg-1 [43]. This parasite was cloned (AS-15CQ) and subjected to further increments in chloroquine pressure to select parasites that survived six daily doses of chloroquine at 30 mg kg-1 [40,42], which were cloned and termed AS-30CQ. Pyrimethamine, chloroquine, mefloquine, artemisinin and artesunate resistance in all parasites was shown to be a stable phenotype after i) freeze/thawing, ii) serial blood passages through mice in the absence of treatment, and iii) transmission through mosquitoes [40-43].

### Drug selection experiments

To select for ATQ resistance, *P. c. chabaudi *clones AS-SENS, AS-PYR, AS-3CQ, AS-15CQ, AS-30CQ, AS-ATN and AS-ART were exposed to gradually increasing concentrations of ATQ during several consecutive passages in mice.

10^7 ^infected red blood cells were diluted in 0.2 ml of citrate saline and inoculated intraperitoneally (i.p.), into groups of ten mice. ATQ at the chosen doses were freshly prepared in pure dimethylsulfoxide (DMSO) and administered orally three hours later. Blood smears were taken every day from day 5 onwards and examined microscopically for parasites. Smears were fixed with methanol and stained in 20% Giemsa for 20 minutes, after which slides were washed and allowed to air-dry. Parasitaemia was calculated as the percentage of infected red blood cells/number of total red blood cells in 10 to 15 microscopic fields. Parasites, which survived drug treatment, were sub-inoculated into fresh mice and the treatment repeated. In subsequent passages, drug concentrations were gradually increased. To discard the possibility that increases in drug tolerance were due to increased virulence caused by multiple sub-inoculations, a parasite line was maintained in parallel and passaged in untreated mice the same number of times as the drug selected line.

### Drug tests

In order to compare the drug responses of drug-selected parasites to those of unselected controls at given time points during the selection procedure, groups of 12 mice were each inoculated i.p. with 10^7 ^parasites (day 0). For this, ten mice were treated orally with drug at various dosages, three hours after parasite inoculation on day 0; the remaining two mice were given DMSO only. ATQ solutions were freshly prepared in DMSO just prior to administration. Blood smears were taken every day from day 6 onwards, stained with Giemsa's stain, and examined microscopically to determine the % parasitaemia in 10 microscopic fields.

### Tests to evaluate the stability of drug-resistance

To assess whether ATQ resistance were a genetically stable feature, drug-resistant parasite clones were re-tested for their drug responses after each of three different procedures: i) freeze-thaw cycles in liquid nitrogen, ii) ten blood sub-inoculations in mice in the absence of drug treatment and iii) transmission through mosquitoes.

Resistance quantification in the drug-selected parasite clones was established in the following way. The minimum curative dose (MCD) of each drug was first assessed in drug-selected parasites and untreated control lines. MCD was defined as the minimum dose of each drug that would prevent re-appearance of parasites in all five mice within each treated group at any time during the first 10 days of the follow-up period. A resistance index was determined using the following equation:

### DNA extraction

Parasitized red cells were harvested from mice under general anaesthesia when trophozoite stages were most prevalent, diluted into citrate saline (pH 7.2) and passed through a column of fibrous cellulose powder twice (CF11^®^, Whatman™) to remove mouse leukocytes [14]. The resulting RBC pellet was washed twice in PBS and parasite DNA extracted by overnight incubation in lysis solution (10 mM Tris [pH 8.0], 50 mM EDTA, 0.1% sodium dodecyl sulfate [SDS], proteinase K [1 mg/ml]) at 42°C. After phenol extraction, DNA was precipitated using propan-2-ol and ammonium acetate (3 M) and dissolved in TE buffer (10 mM Tris-Cl, 1 mM EDTA, pH8.0). DNA samples were stored at -20°C.

### Identification of the P. c. chabaudi cytb gene

The DNA sequences of the *P. falciparum *and *P. yoelii cytb *gene were available online at the NCBI/NIH (National Institute of Health) database [44]. To obtain the *P. c. chabaudi *orthologues of this gene, these sequences were retrieved and used in BLAST searches against the available *P. c. chabaudi *sequences (shot gun clones and genomic contigs), deposited at the *P. c. chabaudi *genome database [45]. The sequences giving significant hits were used to design *P. c. chabaudi*-specific oligonucleotide primers to amplify overlapping DNA fragments spanning the coding region, introns and both 5'- and 3'-non-coding sequences (Table S2, Additional file 1). These were then used in PCR amplifications containing *P. c. chabaudi *genomic DNA templates, as described below.

### Amplification and sequencing of the *cytb *gene of *P. chabaudi*

Genomic DNA was used as template in 50 μl PCR reactions, containing 0.2 μM of each oligonucleotide primer, 1× PCR buffer (PromegaÔ), 2.5 mM MgCl_2_, 0.2 mM dNTPs and 0.025 U/μl of *Taq *DNA polymerase. PCR products were purified using the QIAquickÒ PCR Purification Kit from QIAGEN and sequenced using BigDye chain termination v3.1 (Applied Biosystems). The sequencing reactions were analysed by Macrogen^®^. The primers used in sequencing reactions were those used for the initial amplification of the fragments. Gene and predicted amino-acid sequences were manually compiled, and then compared between drug selected and unselected clones using an internet-based interface denoted Multiple Sequence Alignment with hierarchical clustering [46], and default alignment parameters [47].

## Results and Discussion

### Selection of atovaquone (ATQ) resistant parasites

The *P. chabaudi *parasite lines used were all derived sequentially from a drug-sensitive cloned isolate denoted AS-SENS (see Table S1, Additional file 1 for details). These included: (i) a chloroquine sensitive, pyrimethamine-resistant clone denoted AS-PYR, (ii) a clone exhibiting low-level chloroquine-resistance denoted AS-3CQ, (iii) a clone exhibiting intermediate-level chloroquine-resistance denoted AS-15CQ, (iv) a highly chloroquine-resistant clone denoted AS-30CQ, (v) a clone exhibiting artesunate-resistance denoted AS-ATN and (vi) a clone that is artemisinin-resistant denoted AS-ART.

In order to select *P. chabaudi *clones resistant to ATQ, various parasite lines were subjected initially to sub-curative drug dose. Attempts to establish stable ATQ resistance of the clones named AS-SENS, AS-PYR, AS-15CQ, AS-30CQ, AS-ATN and AS-ART were unsuccessful.

ATQ resistance was only selected from the clone AS-3CQ. Initial drug testing revealed that the maximum dose of ATQ tolerated by ATQ sensitive parent parasites was 20 mg/kg/single dose. Parasites that survived that initial dose were sub-inoculated and subjected to successive increments in drug doses, as described in the Methods section.

AS-3CQ parasite line responded well to the ATQ selection procedure described, and a line that tolerated a daily dose of 120 mg/kg/day, was selected after nine passages. At this stage, it was considered that a significant level of resistance had been successfully achieved and thus the ATQ treatment procedure was suspended.

Parasite lines containing populations of ATQ-resistant parasites were subjected to cloning by limiting dilution. Three clones were successfully established from the ATQ resistant parasite population. Of these, one clone was chosen on the basis of their faster growth rate and designated *P. c. chabaudi *AS-ATQ. AS-ATQ was tested for its response to ATQ, immediately after cloning, and shown to retain the same phenotype as that of the drug-resistant population from which they had been derived. These parasites were then used in subsequent studies to further investigate whether the observed drug resistance was stable.

### ATQ resistance in *P. chabaudi *AS-ATQ is stable

In order to confirm whether an heritable genetic mutation underlies the response of AS-ATQ to ATQ, the stability of the drug resistance phenotype in this clone was evaluated by measuring drug responses after (i) multiple blood passages in the absence of the drug, (ii) freeze/thawing of parasites in liquid nitrogen and (iii) transmission through a mosquito host, *Anopheles stephensi*.

AS-ATQ parasite line was therefore subjected to 10 further passages in untreated mice, after which it was tested for its drug response, it was also deep-frozen in liquid nitrogen, thawed after one week and inoculated back into mice and mice infected with AS-ATQ were used to feed *A. stephensi *mosquitoes which then were used to infect mosquitoes again once the parasite cycle was completed, and tested again for ATQ tolerance. It was observed that, after: a) three cycles of freezing and thawing in liquid nitrogen, b) ten blood sub-inoculations in mice in the absence of drug treatment and c) transmission through mosquitoes, the AS-ATQ line recrudesced on the same day as the parasite line before the tests for drug stability. Thus AS-ATQ resistance has a stable phenotype in the absence of drug, suggesting that the drug resistant phenotype is genetically encoded. The Minimum Curative Dose (MCD) of AS-ATQ was assessed in parallel to the untreated control line, AS-3CQ. AS-ATQ showed a six-fold increase in MCD to ATQ relative to AS-3CQ (See Table S3, Additional file 1 for details).

In summary, ATQ resistance phenotypes of the drug-selected parasite clones were unaltered after passage in the absence of drug pressure, after freezing and thawing and after transmission through laboratory mosquitoes. Therefore, ATQ resistance in clone AS-ATQ is genetically encoded.

### ATQ resistance in *P. chabaudi *is associated with a mutation in the *pccytb *gene

With the objective of investigating whether the *P. chabaudi *orthologue of *Pfcytb *gene played a role in resistance to ATQ in *P. chabaudi*, therefore, *P. chabaudi *specific database sequences were used to design oligonucleotide primers to amplify this gene (See Table S2, Additional file 1 for details).

In order to identify possible point mutations in this gene, in the ATQ-resistant parasites relative to AS-3CQ, the nucleotide sequence of this gene in both sensitive and resistant parasites was determined.

The comparisons of the *Pccytb *coding region between all parasites (AS-SENS, AS-PYR, AS-3CQ, AS-15CQ, AS-30CQ, AS-ART, AS-ATN and AS-ATQ parasites) revealed no differences. A difference was found only on the AS-ATQ sample in relation to its progenitor AS-3CQ, a substitution at the residue Tyr268 for an Asn, this mutation is the homologous of the one found in *P. falciparum *isolates resistant to ATQ [37].

## Conclusions

Overall, the available data indicate that it is possible to select atovaquone (ATQ) resistance using *Plasmodium chabaudi*. ATQ resistance was obtained from a clone that is resistant to pyrimethamine and presents a low resistant phenotype to chloroquine.

Although studies made directly on *P. falciparum *provide more information, this presents several limitations, such as the requirement of working with chimpanzees or humans as hosts for infection, a fact which poses serious ethical problems, which can be circumvented by working with animals models, which make easier the selection of drug-resistant mutants and the identification of genes involved in the resistance.

Of all the rodent malaria models the *Plasmodium chabaudi *model is the most appropriate for studies on the genetics of drug resistance, as *P. chabaudi *is the rodent malaria model that shows most biological similarity to *P. falciparum *because it preferentially parasitizes mature erythrocytes and the schizogony is synchronous. In addition several clones of this species are already available, which have been selected for resistance to a variety of different drugs. These parasites present the ideal starting material for identifying drug resistance genes since they have been selected from cloned sensitive parasites. In this way, the mutant parasites should have identical genetic backgrounds to the starting sensitive forms except for the genes determining resistance.

Having all this in mind our objective was to determine the ATQ sensitivity of the *P. chabaudi *clones and thus to select a resistant parasite line and also to perform genotypic characterization of the *cytb *gene of these clones. The objective of selecting an ATQ drug resistant clone of *P. chabaudi *of stable phenotype was fulfilled and this new clone can in the future be use as a model for future studies on malaria combined therapy with ATQ being one of the drugs in the combination.

The study presented here gives extra evidence of the genetic similarities between *P. chabaudi *and *P. falciparum *concerning the genetics of drug resistance, as the mutation detected in the *Pccyb *gene from the AS-ATQ resistant clone is the same as the most common mutation detected in *Pfcytb *gene in *P. falciparum*.

## Competing interests

The authors declare that they have no competing interests.

## Authors' contributions

AA did the sequencing of the clones, was responsible for data collection and drafted the manuscript. VDR was responsible for coordination of laboratory and project.

AA, ZN and DL performed all PCR amplifications and actively participated in sample collection. AA, ZN, ACA, HC established suitable protocols for *Pfcytb *amplification and participated in drafting the manuscript. AA and ZN were responsible for the sequencing procedure. AMT conceived the study, helped for sequence analysis, drafted and revised the manuscript. All authors read and gave the final approval of the version to be published.

## Supplementary Material

Additional file 1 **Table S1**. Clones of *Plasmodium chabaudi *and the procedure use for their selection. Click here for file
