# Supplementary material for: Plasmodium chabaudi chabaudi malaria parasites can develop stable resistance to atovaquone with a mutation in the cytochrome b gene
Source: Malar J. 2010 May 21;9:135. doi: 10.1186/1475-2875-9-135 (PMC2881937; doi:10.1186/1475-2875-9-135)
Supplement: Additional file 1 — Table S1. Clones of Plasmodium chabaudi and the procedure use for their selection. [file 1475-2875-9-135-S1.PDF]

Table S1 – Clones of *Plasmodium chabaudi* and the procedure use for their selection.

CLONE	DRUG RESPONSE
AJ	Drug sensitive, genetically different from AS
AS (SENS)	Drug sensitive
AS (PYR)	Selected from AS (sens); pyrimethamine-resistant
AS (3 CQ)	Selected from AS (pyr); low chloroquine-resistant
AS (15 CQ)	Selected from AS (3CQ); intermediate chloroquine-resistant
AS(ATN)	Selected from AS(15CQ); artesunate-resistant
AS (30 CQ)	Selected from AS (15CQ); high chloroquine-resistant
AS(ART)	Selected from AS(30CQ); artemisinin-resistant

Table S2 - Polymerase Chain Reactions for sequencing of *Pccytb* gene.

Gene	Primer sequence (5'-3´)	$[MgCl_2]$/ [Primer]
	sense – GACGCTTTAAATGGGTGGAA	3 mM / 0.8 μM
	antisense - TTCTGGAGAATAACGACTAGCTAAGA	
	sense – TGGAGTGGATGGTGC	4.5 mM / 0.8 μM
	antisense – TCCTGAAATCCATGATAAAGGT	
Pccyt	sense – GCTACTGGTGCATCACTTGTATTC	4.5 mM / 0.8 μM
	antisense – TTGCACCCCAATAACTCATTT	
	sense – AAATGAGTTATTGGGGTGCAA	3.5 mM / 0.4 μM
	antisense – CCCTAAAGGATTAGTGCTACCAT	
	sense – GGTAGCACTAATCCTTTAGGGTATGA	4.5 mM / 0.8 μM
	antisense – GCATTATCTGGATGTGATAATGGTA	
	sense – CATTATCACATCCAGATAATGCAA	4.5 mM / 0.8 μM
	antisense – CGAATTGAAGTGTGGAGAGAA	
	sense – TGGCATCTCTCTCGATTTACAG	4.5 mM / 0.8 μM
	antisense – TCGATATACGGATTTCTCCTGAA	

PCR amplification conditions: 40 cycles. 95ºC for 600'', 95ºC with a 0' hold, cooling at 20ºC/s to 63ºC with a 7' hold, heating at 20ºC/s to 72ºC with a 7' hold. Heating at 20ºC/s to 95ºC with 0' hold, cooling at 20ºC/s at 65ºC and heating at 0.2ºC/s to 95ºC.

Table S3 - N-fold resistance of *P. chabaudi chabaudi* AS-ATQ.

P. chabaudi	MCD ATQ (mg/Kg/single dose)	N-fold ATQ
AS-SENS	20	-----
AS-ART	20	-----
AS-3CQ	20	-----
AS-ATQ	120	6

The absolute and relative (N-fold) drug sensitivities of AS-ATQ after blood passage in the absence of treatment, freeze/thaw and mosquito transmission are given.

MCD – minimum curative dose
